# Impact of perioperative steroid administration in patients undergoing elective liver resection: meta-analysis

**DOI:** 10.1093/bjsopen/zrac139

**Published:** 2022-12-20

**Authors:** Laila Jötten, Kira C Steinkraus, Benno Traub, Sandra Graf, André L Mihaljevic, Marko Kornmann, Christoph W Michalski, Felix J Hüttner

**Affiliations:** Department of General and Visceral Surgery, Ulm University Hospital, Ulm, Germany; Department of General and Visceral Surgery, Ulm University Hospital, Ulm, Germany; Department of General and Visceral Surgery, Ulm University Hospital, Ulm, Germany; Department of General and Visceral Surgery, Ulm University Hospital, Ulm, Germany; Department of General and Visceral Surgery, Ulm University Hospital, Ulm, Germany; Department of General and Visceral Surgery, Ulm University Hospital, Ulm, Germany; Department of General and Visceral Surgery, Ulm University Hospital, Ulm, Germany; Department of General and Visceral Surgery, Ulm University Hospital, Ulm, Germany

## Abstract

**Background:**

Perioperative steroid administration may improve postoperative outcomes in major abdominal surgery by reducing the systemic inflammatory response. The aim of this systematic review was to evaluate the impact of perioperative steroid administration on outcomes after elective liver resection.

**Methods:**

PubMed, Cochrane Library, and Web of Science were systematically searched for randomized clinical trials (RCTs) comparing perioperative steroid administration with placebo, standard of care, or no steroids with respect to postoperative outcomes, particularly postoperative complications. Two independent reviewers critically appraised the studies and extracted data. Meta-analyses were performed using a random-effects model with ORs calculated for dichotomous outcomes and mean differences (MDs) for continuous outcomes.

**Results:**

Ten RCTs comprising 930 patients were included. Perioperative steroid administration significantly reduced the overall postoperative complication rate (OR 0.61, 95 per cent c.i. 0.43 to 0.87; *P* = 0.006; *I*^2^ = 26 per cent). No significant differences were shown for individual complications. Several postoperative laboratory parameters were positively affected, like total serum bilirubin (MD −0.46; 95 per cent c.i. −0.74 to −0.18; *P* = 0.001; *I*^2^ = 80 per cent), interleukin 6 (MD −48.99; 95 per cent c.i. −60.72 to −37.27; *P* < 0.001; *I*^2^ = 0 per cent) and C-reactive protein (MD −5.20; 95 per cent c.i. −7.62 to −2.77; *P* < 0.001; *I*^2^ = 71 per cent). There were no signs of an increase in potential steroid-induced adverse events, namely infectious complications, thromboembolic events, or bleeding.

**Conclusions:**

Perioperative steroid administration significantly reduces the overall complication rate after elective liver resection without an increased risk of adverse effects.

## Teaser

In this manuscript, we performed a meta-analysis of randomized clinical trials investigating the impact of perioperative steroid administration surrounding elective liver resections. The results based on a total of 930 patients demonstrated a positive effect of steroid administration on overall postoperative complications and several laboratory parameters of liver function and inflammation.

## Introduction

Despite continuous improvements in surgical technique and perioperative care, elective liver resections are still associated with significant postoperative morbidity deaths and mortality deaths^1–4^. Major abdominal surgery induces physical stress, triggering a systemic inflammatory response mainly via mediators such as interleukin (IL)-1, tumour necrosis factor (TNF-α), and IL-6^5–11^. Elevated postoperative IL-6 levels after major abdominal surgery are associated with an increased rate of complications and length of hospital stay^12–14^. One of the most important complications after liver resections is posthepatectomy liver failure (PHLF)^15,16^. Furthermore, hepatic pedicle clamping (Pringle manoeuvre), which is intended to minimize blood loss during hepatectomy, may cause increased oxidative stress, an intensified systemic inflammatory response, and incremental liver function impairment ^17–20^. Total serum bilirubin (TSB) and prothrombin time (PT, international normalized ratio (INR)) have been shown to be useful predictors of postoperative liver failure and mortality deaths^15,16^.

A variety of approaches aimed at reducing complications after liver resection have been already tested. Surgical modifications include selective or intermittent venous clamping instead of continuous to minimize ischaemia–reperfusion injury^21–23^. Furthermore, various pharmaceutical interventions, such as dextrose, erythropoietin, trimetazidine, ulinastatin, or vitamin E have been used and many others are currently in preclinical testing^19,22,24–26^. There are also various studies examining perioperative steroid administration in liver resection, including several randomized clinical trials (RCTs) that, however, led to conflicting results^27–31^. A positive effect on postoperative complications may be possibly explained by the inhibition of systemic inflammation and oxidative stress by reduction of inflammatory mediators^32–34^. On the other hand, the safety profile of perioperative steroid administration, particularly regarding delayed wound healing, infectious complications, thromboembolic events, and gastrointestinal bleeding needs to be considered^35–37^. Based on the recent publication of additional RCTs on this topic^27,30^, an up-to-date systematic review and meta-analysis elucidating the potential risks and benefits of perioperative steroid treatment in hepatic surgery is needed.

## Methods

This systematic review was conducted in accordance with the recommendations of the PRISMA guidelines^38^. Study selection, critical appraisal and data extraction were independently performed by two reviewers. Disagreement was resolved by discussion and/or a joint re-assessment of the primary studies. The review was prospectively registered with PROSPERO (CRD42022336117).

### Systematic literature search

The electronic databases PubMed, the Cochrane Library, and Web of Science were systematically searched up to 31 January 2022 for RCTs comparing perioperative steroid treatment with placebo, no treatment, or standard of care (such as postoperative nausea prophylaxis with low-dose steroids), in patients undergoing liver resection^39^. The were no restrictions on language or publication date. The Cochrane sensitivity and precision maximizing RCT filter was used for the search. Reference lists of relevant studies and related systematic reviews were manually screened to further broaden the search. The final search strategies for all databases are provided in the *Supplementary material* of this article (*Appendixes S1–S3*). Search strategies for the Cochrane Library and Web of Science were adapted to the specific vocabulary of the respective database.

### Study selection

RCTs meeting all the following inclusion criteria were eligible: Adult patients of all ages and sexes; any elective liver resection, including minor and major resections, to treat any underlying disease; and comparison of perioperative steroid treatment with placebo treatment, standard of care, or no steroid treatment at all. Perioperative intervention was defined as administration of the appropriate substance a few hours before and/or throughout the surgery. Non-randomized trials, animal trials, and trials that applied additional interventions in one of the two groups were excluded. In trials with more than two arms, only the arms including steroid treatment and placebo/standard of care/no steroid treatment were extracted.

Titles and abstracts retrieved by the systematic literature search were screened by two independent reviewers. The full text was obtained if either one of the reviewers considered that the article was potentially eligible. Full texts were assessed in detail for final inclusion in this systematic review.

### Outcome measures

The primary endpoint was overall postoperative complication rate. Secondary outcome measures were mortality deaths, length of postoperative hospital stay, individual complications (PHLF preferably defined according to the definition of the International Study Group of Liver Surgery^40^, overall infectious complications, surgical site infection (SSI), bile leakage, pleural effusion, bleeding, thromboembolic events and reoperation), postoperative mortality deaths, postoperative liver function tests (TSB, INR, aspartate aminotransferase (AST), alanine aminotransferase (ALT)), and postoperative inflammatory markers (C-reactive protein (CRP) and IL-6). For all laboratory parameters, the postoperative peak value was considered.

### Data extraction

Two reviewers independently extracted the data using a predefined electronic data extraction sheet. These included study characteristics, baseline data of included patients, quality characteristics, and the outcome measures described above for the individual treatment groups. Liver resections were defined as major if three or more segments were resected. When several reports of the same study population existed, the most comprehensive publication was evaluated and was supplemented by additional data of the secondary publications, if reasonable and necessary. If data were not reported in sufficient detail or other information was lacking, the corresponding authors of the primary publications were contacted for clarification or a request for additional data.

### Critical appraisal

Critical appraisal of the methodological quality of the included studies was assessed by two reviewers independently using the Cochrane risk-of-bias tool^41^. To judge the overall certainty of the synoptic evidence, the GRADE approach was used^42^. For assessment of publication bias, a funnel plot was created for the primary outcome.

### Statistical analysis

All analyses were performed using R version 4.0.0. Meta-analyses were performed for the above-mentioned endpoints, if three or more trials reported data for a specific outcome. For dichotomous outcomes, ORs were calculated using the Mantel–Haenszel method. As an exception, the risk difference (RD) was calculated by the Mantel–Haenszel method for the endpoint ‘mortality deaths’ in anticipation of a number of trials with zero events in both groups. For continuous outcomes, mean differences (MDs) were calculated using the inverse variance method. Summary effect measures were presented along with their corresponding 95 per cent confidence intervals. A random-effects model was chosen for the meta-analyses to account for clinical heterogeneity between trials. A sensitivity analysis was performed including only trials with a placebo control. Furthermore, the following subgroup analysis were performed for the primary endpoint: trials with at least one judgement of ‘high risk of bias’ *versus* trials without any ‘high risk of bias’ judgement and trials that used methylprednisolone *versus* trials that applied hydrocortisone. The Harbord test was performed to examine funnel plot asymmetry^43^. The method of Hozo *et al*.^44^ was used to estimate mean(s.d.) for trials that reported medians and ranges only. For median and interquartile range data, calculations were performed using the method of Wan *et al*.^45^. Statistical heterogeneity was assessed using the *I*^2^ statistic.

## Results

**Fig. 1:**
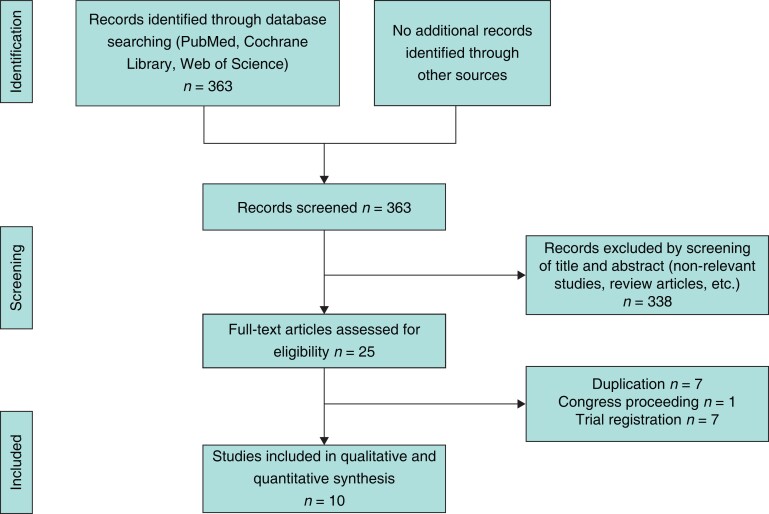
Flow chart of study selection

The literature search in the electronic databases yielded 363 hits. After further stepwise screening for eligibility, 10 RCTs reported in 17 publications were finally included in the present systematic review and meta-analysis^27–30,46–51^. The study selection process is depicted in a flow chart (*Fig. 1*).

The trials were conducted in Canada, Denmark, Germany, Italy, and Japan. The RCTs included a cumulative cohort of 930 patients who underwent elective liver resection. Of these, 469 (50.4 per cent) patients received perioperative steroids and 461 (49.6 per cent) did not. The most common surgical indication were liver metastases (*n* = 368; 39.6 per cent), followed by hepatocellular carcinoma (*n* = 285; 30.6 per cent), and cholangiocarcinoma (*n* = 128; 13.8 per cent). In the entire cohort, 446 patients (48.0 per cent) underwent major hepatectomies with balanced distribution between both groups. Two trials did not report exactly in how many patients the Pringle manoeuvre was used; one stated that it was applied in ‘nearly all’ patients and the other in ‘almost all’ patients^47,51^. In the remaining 697 patients, an intermittent or continuous Pringle manoeuvre was performed in 455 patients (65.3 per cent) with similar distribution in the steroid and control groups. Additional clinical characteristics of patients were also comparable within the two groups (namely age, sex, disease, extent of resection, Pringle manoeuvre, and cirrhosis; *Tables 1* and *2*). Perioperative administration of methylprednisolone occurred in eight and hydrocortisone in two of the 10 RCTs. The detailed intervention schemes are shown in *Table 1*. All trials involved preoperative steroid administration, whereas Hayashi *et al*.^51^ and Onoe *et al*.^30^ involved additional steroid administrations on postoperative day (POD) 1, 2, and 3 with reduced doses (*Table 1*). In the trial by Steinthorsdottir *et al*.^31^, patients in the control group received the standard of care (8 mg of dexamethasone as postoperative nausea prophylaxis), whereas the intervention group received 10 mg/kg prednisolone after induction of anaesthesia. No information regarding the definition of PHLF was available for Hayashi *et al*.^51^, Steinthorsdottir *et al*.^31^, and Yamashita *et al*.^47^. Furthermore, Aldrighetti *et al.*^28^ characterized PHLF by the presence of encephalopathy, ascites of more than 500 ml per day, INR more than 2 or more than 1.5 on POD5, or bilirubin levels of more than 3 mg/dl on POD5.

**Table 1:** Trial characteristics

Trial/reference	Country of origin	No. of patients	Age (years)	Sex ratio (M : F)		Interventions
		S/C	S/C	F S/C	M S/C	
Aldrighetti *et al*.^28^	Italy	36/37	61.8(14.3)/63(13.5)	14/14	22/23	Steroid group: 500 mg methylprednisolone at induction of anaesthesia Control group: not specified
Bressan *et al*.^27^	Canada	77/74	63.9(9.4)/62.4(9.4)	30/35	47/39	Steroid group: 500 mg methylprednisolone at induction of anaesthesia Control group: placebo (saline)
Donadon *et al*.^48^	Italy	16/16	59.3(13.3)/56.3(13.8)	6/7	10/9	Steroid group: 500 mg methylprednisolone as bolus before hepatectomy, followed by 500 mg methylprednisolone as continuous infusion for approximately 6 h Control group: placebo (glucose 5%)
Hasegawa *et al*.^29^	Japan	50/50	66.7(11.5)/68.3(9.9)	20/19	30/31	Steroid group: 500 mg methylprednisolone at induction of anaesthesia Control group: placebo (saline)
Hayashi *et al*.^51^	Japan	102/98	69(7)/70(7.8)	NR	NR	Steroid group: 500 mg hydrocortisone immediately before hepatic pedicle clamping, 300 mg on POD1, 200 mg on POD 2, 100 mg on POD3 Control group: placebo (saline)
Muratore *et al*.^50^	Italy	25/28	65.4(10.8)/64.1(11.7)	8/17	17/11	Steroid group: 30 mg methylprednisolone per kg bodyweight 30 min before hepatectomy Control group: not specified
Onoe *et al*.^30^	Japan	48/46	70(11)/71(11.3)	19/15	29/31	Steroid group: 500 mg hydrocortisone immediately before pedicle clamping, 300 mg on POD1, 200 mg on POD2, and 100 mg on POD3 Control group: placebo (saline)
Schmidt *et al*.^49^	Germany	10/10	57/65	7/6	3/4	Steroid group: 30 mg methylprednisolone per kg bodyweight 90 min before surgery Control group: placebo (saline)
Steinthorsdottir *et al*.^31^	Denmark	88/86	65.2(11.2)/64.4(12.0)	27/30	61/56	Steroid group: 10 mg methylprednisolone per kg bodyweight approximately 30 min before surgery Control group: standard of care (8 mg dexamethasone as postoperative nausea prophylaxis)
Yamashita *et al*.^47^	Japan	17/16	60.3(10.3)/56.8(22.4)	4/7	13/11	Steroid group: 500 mg of methylprednisolone 2 h before surgery Control group: not specified

Values are mean(s.d.). All studies were randomized clinical trials. C, control group; NR, not reported; POD, postoperative day; S, steroid group.

**Table 2:** Surgical characteristics

Trial/reference	Pathology		Resection		Pringle manoeuvre	Cirrhosis
		S/C	minor S/C	major S/C	S/C	S/C
Aldrighetti *et al*.^28^	Metastasis	24/26	12/13	24/24	36/37	14/12
	HCC	14/14				
	CCA	4/4				
	GB cancer	0/1				
	BD	3/3				
	HAE	1/1				
Bressan *et al*.^27^	Metastasis	61/54	0/0	77/74	12/15	4/4
	HCC	11/16				
	CCA	5/4				
Donadon *et al*.^48^	Metastasis	6/12	9/11	7/5	16/16	0/0
	HCC	6/2				
	CCA	4/0				
	Others	0/2				
Hasegawa *et al*.^29^	HCC	23/26	40/39	10/11	50/50	8/8
	Metastasis	21/14				
	Others	6/10				
Hayashi *et al*.^51^	HCC	63/66	91/83	11/15	NR/NR	3/4
	Metastasis	32/33				
	CCA	6/5				
	Others	1/4				
Muratore *et al*.^50^	NR	NR/NR	12/13	13/15	25/28	7/9
Onoe *et al*.^30^	CCA	43/41	0/0	48/46	48/46	0/0
	GB cancer	2/3				
	BD	2/1				
	HCC	1/1				
Schmidt *et al*.^49^	Metastasis	4/4	4/5	6/5	0/0	0/0
	BD	4/3				
	HCC	2/1				
	CCA	0/2				
Steinthorsdottir *et al*.^31^	Metastasis	66/64	67/63	21/23	41/35	7/9
	HCC	10/10				
	BD	3/8				
	CCA	6/3				
	GB cancer	0/1				
	Others	3/0				
Yamashita *et al*.^47^	HCC	13/8	12/10	5/6	NR/NR	NR/NR
	LLTD	4/4				
	Metastasis	0/3				
	CCA	0/1				

Minor resection, fewer than three segments; major resection, three or more segments; C, control groups; NR, not reported; S, steroid group; BD, benign disease; CCA, cholangiocarcinoma; GB, gall bladder; HAE, haemangioendothelioma; HCC, hepatocellular carcinoma; LLTD, living liver transplant donor.

### Critical appraisal

**Fig. 2:**
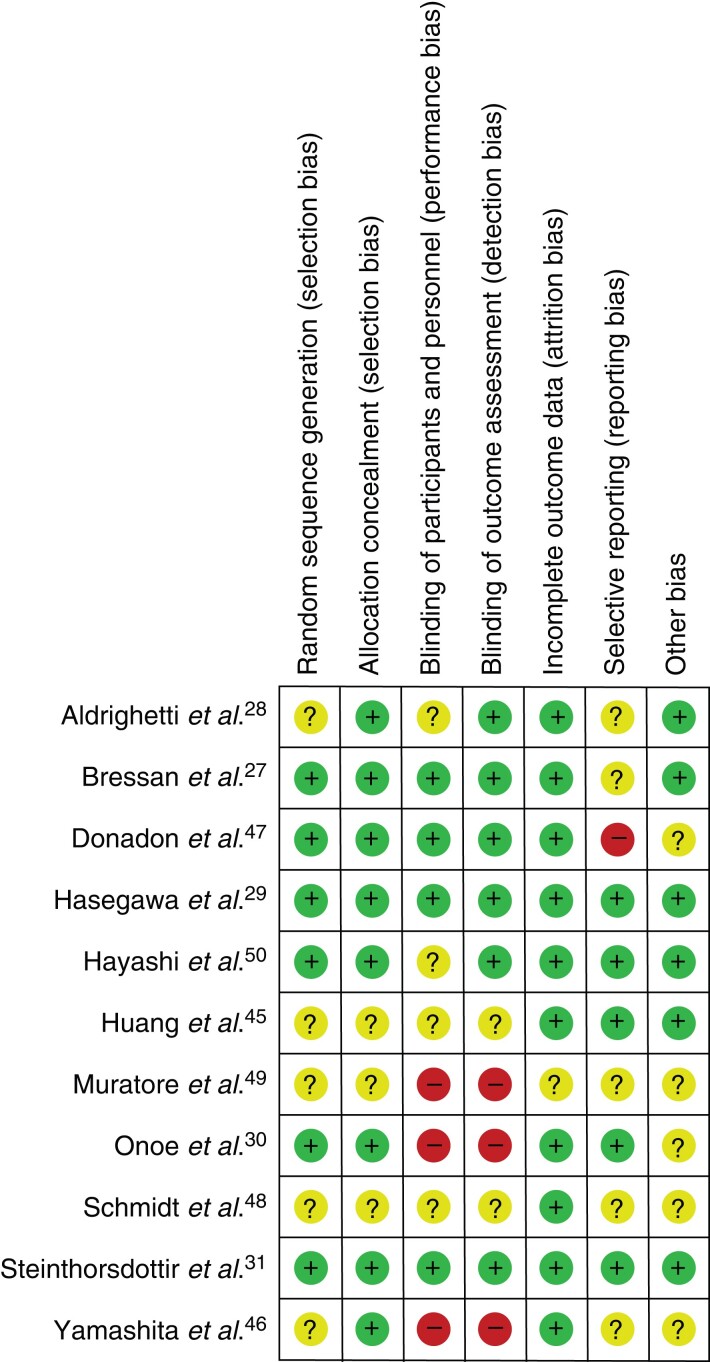
Risk of bias summary graph

*Figure 2* provides an overview of the risk of bias assessment. All trials were monocentric. Only two trials were judged to be at low risk of bias in all domains^29,31^. Regarding the trials and domains that were judged to present a potentially high risk of bias, there were ambiguities between the endpoints in the trial registration and the published results in the trial of Donadon *et al*.^48^, so that a reporting bias could not be excluded. Muratore *et al*.^50^, Onoe *et al.*^30^, and Yamashita *et al.*^47^ did not blind the participants/personnel or the outcome assessors. Thus, the risk of performance and detection bias was judged as high in these trials.

### Overall postoperative complications

**Fig. 3:**
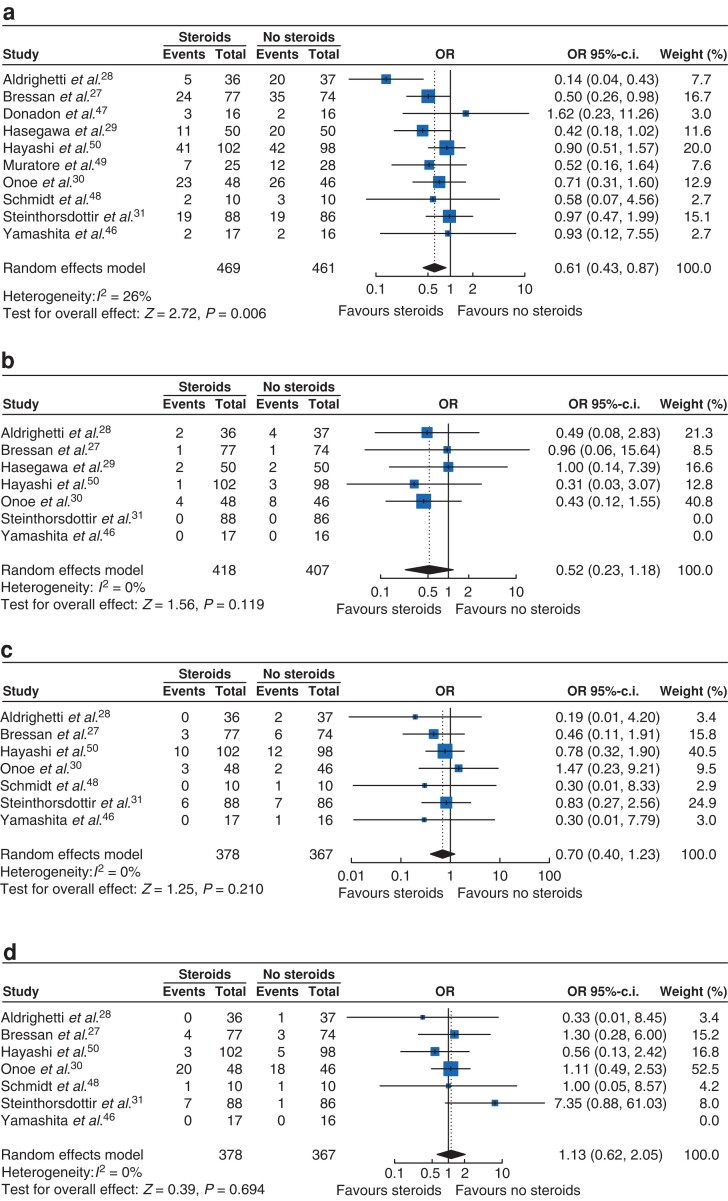
Forest plots of postoperative complications **a** Overall complications. **b** Post-hepatectomy liver failure. **c** SSI (superficial). **d** Bile leakage. ORs calculated by the Mantel–Haenszel method. Summary effect measures are shown along with corresponding 95 per cent confidence intervals, random-effects model.

The overall postoperative complication rate was 29.2 per cent (137 of 469 patients) in the steroid group and 39.3 per cent (181 of 461 patients) in the control group. Meta-analysis demonstrated a significant difference between the two groups in favour of the steroid group (10 trials, OR 0.61, 95 per cent c.i. 0.43 to 0.87; *P* = 0.006; *I*^2^ = 26 per cent). Thus, the rate of overall complications could be reduced by approximately 10 per cent by steroid administration, resulting in a number needed to treat of 10. The results of the meta-analysis are illustrated in *Fig. 3a*. The associated funnel plot, showing no relevant risk for publication bias, can be found in the *Supplementary material* (*Fig. S1*). The test of funnel plot asymmetry showed no statistical significance (*t* = −0.22, 8 d.f., *P* = 0.829).

The sensitivity analysis including only trials with a placebo control, confirmed the results of the primary analysis with a statistically significant result (six trials, OR 0.67, 95 per cent c.i. 0.48 to 0.94; *P* = 0.019; *I*^2^ = 0 per cent). Both subgroup analyses did not show any significant subgroup differences and the direction of the effect remained the same in all subgroups. While the subgroup of trials without any judgement of ‘high risk of bias’ corroborated the statistically significant result for the primary endpoint (six trials, OR 0.54, 95 per cent c.i. 0.32 to 0.93; *P* = 0.026; *I*^2^ = 54 per cent), the subgroup of trials with at least one judgement of ‘high risk of bias’ did not show statistical significance (four trials, OR 0.72, 95 per cent c.i. 0.39 to 1.31; *P* = 0.284; *I*^2^ = 0 per cent). Similarly, the subgroup of trials that used methylprednisolone confirmed the primary results (eight trials, OR 0.53, 95 per cent c.i. 0.33 to 0.85; *P* = 0.009; *I*^2^ = 29 per cent), whereas the subgroup using hydrocortisone demonstrated no statistical significance (two trials, OR 0.83, 95 per cent c.i. 0.52 to 1.32; *P* = 0.430; *I*^2^ = 0 per cent).

### Secondary outcomes

#### Individual complications

Meta-analyses of individual complications showed no differences for PHLF (seven trials, OR 0.52, 95 per cent c.i. 0.23 to 1.18; *P* = 0.119; *I*^2^ = 0 per cent; *Fig. 3b*), bile leakage (seven trials, OR 1.13, 95 per cent c.i. 0.62 to 2.05; *P* = 0.694; *I*^2^ = 0 per cent; *Fig. 3d*), superficial SSIs (seven trials, OR 0.70, 95 per cent c.i. 0.40 to 1.23; *P* = 0.210; *I*^2^ = 0 per cent; *Fig. 3c*) and organ space SSIs (five trials, OR 1.17, 95 per cent c.i. 0.49 to 2.81; *P* = 0.717; *I*^2^ = 50 per cent; *Fig. S2*). Similarly, no significant differences were shown for pleural effusion (four trials, OR 0.93, 95 per cent c.i. 0.48 to 1.80; *P* = 0.828; *I*^2^ = 0 per cent; *Fig. S3*), and reoperations (four trials, OR 0.63, 95 per cent c.i. 0.27 to 1.50; *P* = 0.299; *I*^2^ = 0 per cent; *Fig. S4*).

#### Safety endpoints

Mortality deaths (eight trials, RD 0.00, 95 per cent c.i. −0.01 to 0.01; *P* = 0.883; *I*^2^ = 0 per cent; *Fig. S5*) and bleeding complications (three trials, OR 0.50, 95 per cent c.i. 0.14 to 1.78; *P* = 0.283; *I*^2^ = 0 per cent; *Fig. S6*) did not differ significantly between both groups. The rate of overall postoperative infectious complications was only reported by Aldrighetti *et al*.^28^, with a significantly lower rate in the steroid group, and Onoe *et al*.^30^ without a significant difference between both groups. Similarly, thromboembolic events were only reported in the trials by Bressan *et al*.^27^ and Steinthorsdottir *et al*.^31^ showing no significant differences in both trials.

#### Perioperative outcomes

Perioperative steroid administration did not relevantly affect length of postoperative hospital stay (10 trials, MD −0.63 days, 95 per cent c.i. −1.44 to 0.17; *P* = 0.122; *I*^2^ = 54 per cent; *Fig. S7*), duration of surgery (nine trials, MD −8.93 min, 95 per cent c.i. −18.41 to 0.55; *P* = 0.065; *I*^2^ = 12 per cent; *Fig. S8*), and intraoperative blood loss (10 trials, MD 16.36 ml, 95 per cent c.i. −23.57 to 56.30; *P* = 0.422; *I*^2^ = 39 per cent; *Fig. S9*).

#### Laboratory parameters

**Fig. 4:**
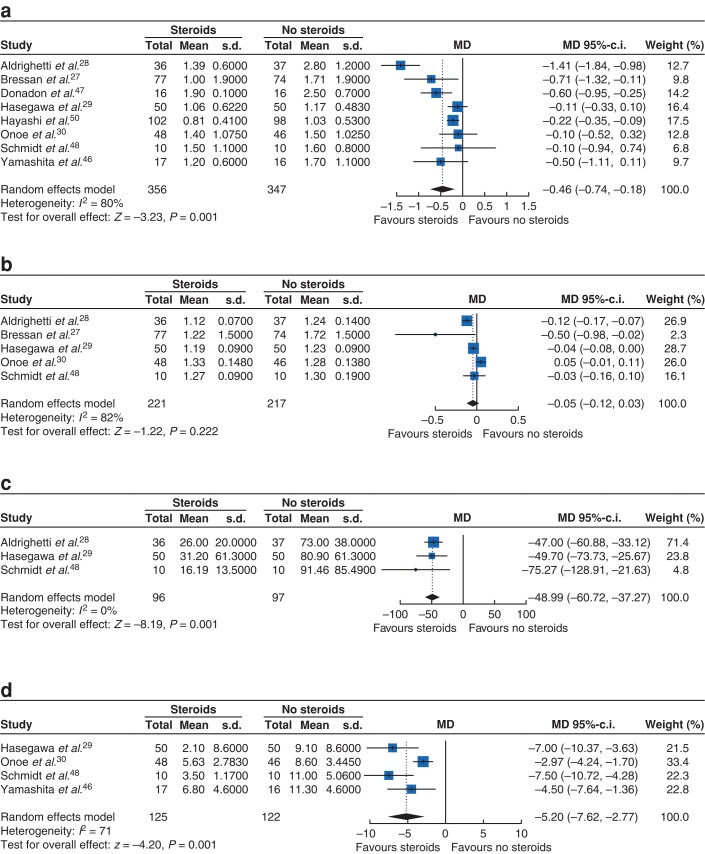
Forest plots of laboratory parameters **a** Total serum bilirubin. **b** Prothrombin time. **c** Interleukin-6. **d** C-reactive protein. MDs were calculated by the inverse variance method. Summary effect measures are shown along with corresponding 95 per cent confidence intervals, random-effects model. MD, mean difference.

Considering the postoperative liver function tests, steroid administration significantly reduced the postoperative peak TSB level by 0.46 mg/dl (eight trials, 95 per cent c.i. −0.74 to −0.18; *P* = 0.001; *Fig. 4a*), but there was substantial statistical heterogeneity in the meta-analysis (*I*^2^ = 80 per cent). There was no relevant effect of perioperative steroid administration on the postoperative values for INR (five trials, MD −0.05, 95 per cent c.i. −0.12 to 0.03; *P* = 0.222; *I*^2^ = 82 per cent; *Fig. 4b*), AST (five trials, MD −7.92 U/l, 95 per cent c.i. −159.40 to 143.56; *P* = 0.918; *I*^2^ = 74 per cent; *Fig. S10*), and ALT (five trials, MD 3.76 U/l, 95 per cent c.i. −130.32 to 137.84; *P* = 0.956; *I*^2^ = 70 per cent; *Fig. S11*) also with substantial statistical heterogeneity in the results. In terms of inflammatory parameters, postoperative peak IL-6 (three trials, −48.99 pg/dl, 95 per cent c.i. −60.72 to 37.27; *P* < 0.001; *I*^2^ = 0 per cent; *Fig. 4c*) as well as postoperative CRP values (four trials, −5.20 mg/dl, 95 per cent c.i. −7.62 to 2.77; *P* < 0.001; *I*^2^ = 71 per cent; *Fig. 4d*) were significantly reduced in the steroid group. Unfortunately, the exact laboratory values for some of the RCTs were not obtained and the authors of the primary trials did either not answer our requests or were not able to provide further detailed data.

### Certainty of the resulting evidence

A summary of findings table was created rating the certainty of the resulting evidence by the GRADE approach^42^. There is moderate certainty evidence that perioperative steroid administration reduces overall complications and IL-6, while there is no relevant effect on PHLF and superficial SSIs. The certainty of the evidence for all other outcomes had to be downgraded to low or very low due to limitations in terms of inconsistency or imprecision (*Table 3*).

**Table 3:** Summary of findings/GRADE assessment

Certainty assessment							No. of patients		Effect		Certainty
No. of trials	Study design	Risk of bias	Inconsistency	Indirectness	Imprecision	Other considerations	Steroids	No steroids	Relative (95% c.i.)	Absolute (95% c.i.)	
**Overall complications**											
10	RCTs	Serious*	Not serious	Not serious	Not serious	None	137/469 (29.2%)	181/461 (39.3%)	OR 0.61 (0.43 to 0.87)	110 fewer per 1.000 (from 175 fewer to 33 fewer)	⨁⨁⨁◯ Moderate
**Post-hepatectomy liver failure**											
7	RCTs	Serious*	Not serious	Not serious	Not serious	None	10/418 (2.4%)	18/407 (4.4%)	OR 0.52 (0.23 to 1.18)	21 fewer per 1.000 (from 34 fewer to 8 more)	⨁⨁⨁◯ Moderate
**SSI (wound)**											
7	RCTs	Serious*	Not serious	Not serious	Not serious	None	22/378 (5.8%)	31/367 (8.4%)	OR 0.70 (0.40 to 1.23)	24 fewer per 1.000 (from 49 fewer to 17 more)	⨁⨁⨁◯ Moderate
**Intraoperative blood loss**											
10	RCTs	Serious*	Serious†	Not serious	Not serious	None	469	461	–	MD 16.36 higher (23.57 lower to 56.3 higher)	⨁⨁◯◯ Low
**Postoperative peak bilirubin**											
8	RCTs	Serious*	Serious‡	Not serious	Not serious	None	356	347	–	MD 0.46 lower (0.74 lower to 0.18 lower)	⨁⨁◯◯ Low
**Postoperative peak IL-6**											
3	RCTs	Serious*	Not serious	Not serious	Not serious	None	96	97	–	MD 48.99 lower (60.72 lower to 37.27 lower)	⨁⨁⨁◯ Moderate
**Postoperative peak CRP**											
4	RCTs	Serious*	Serious§	Not serious	Not serious	None	125	122	–	MD 5.2 lower (7.62 lower to 2.77 lower)	⨁⨁◯◯ Low

MD, mean difference; RCT, randomized clinical trial; SSI, surgical site infection; IL, interleukin; CRP, C-reactive protein. *Primary trials present some potential sources of bias (for example lack of blinding and differing outcome definitions), which lower the confidence in the results. †Inconsistent direction of effects in between trials. ‡. Relevant inconsistency with high *I*^2^ (80 per cent), not overlapping 95 per cent c.i., but consistent direction of effect. §Relevant inconsistency with high *I*^2^ (71 per cent), not overlapping 95 per cent c.i., but consistent direction of effect.

## Discussion

This systematic review and meta-analysis examined the risk and benefit of perioperative steroid administration in elective liver resection, including two recently published RCTs on this topic^52,53^. The aggregated data demonstrate a significant and clinically relevant benefit of the intervention in terms of a reduced postoperative overall complication rate as well as several laboratory parameters of liver function (TSB) and postoperative inflammation (IL-6 and CRP). The validity of the results was confirmed in several subgroup and sensitivity analyses. There were no negative side effects of steroid administration, in particular no increased risk of infectious, thromboembolic, or bleeding complications. The underlying evidence is based on 10 RCTs with a total of 930 patients. Nevertheless, applying the GRADE approach, the certainty of evidence had to be downgraded for all endpoints due to some potentially relevant risk of bias. Thus, the certainty of evidence for the primary outcome is moderate.

The perioperative administration of corticosteroids has been shown to reduce postoperative complications after other major abdominal surgeries, such as pancreatic resections^54^. The most likely underlying mechanism for these results seems to be the beneficial effect of steroid administration on the physical stress and associated systemic inflammatory response induced by major abdominal surgery^5,7,9,11^. This hypothesis is supported by the current results regarding postoperative inflammatory parameters; however, the power of the results regarding inflammatory parameters is limited by the smaller number of RCTs that could be included in these meta-analyses. In corroboration of the meta-analyses, Hayashi *et al*.^51^, Muratore *et al*.^50^, and Yamashita *et al.*^47^ also reported a significant reduction of postoperative IL-6 in the steroid administration group. Unfortunately, it was not possible to obtain the exact values of these trials, so inclusion in the meta-analysis was not possible.

In the current meta-analysis, no significant reduction of individual complications such as PHLF, infections, bile leakage, pleural effusion, bleeding, or reoperation was shown. Unfortunately, only seven of 10 RCTs reported on PHLF and even fewer RCTs reported on other individual complications, so that the statistical power was limited for these endpoints. In this context, it seems reasonable to assess postoperative liver function parameters, as for example TSB and PT (INR) have been shown in previous studies to be useful predictors of postoperative liver failure^15,16^. In the present meta-analysis, although there was a significant decrease in postoperative peak of serum bilirubin, there was no effect on postoperative peak of INR, AST, and ALT. Again, these results are limited by the smaller number of RCTs included in the meta-analysis. Hayashi *et al*.^51^, Muratore *et al*.^50^, Steinthorsdottir *et al*.^31^, and Yamashita *et al*.^47^ also tested postoperative prothrombin time and Muratore *et al*.^50^ and Steinthorsdottir *et al*.^31^ even reported a significant difference, favouring steroid administration. Unfortunately, it was not possible to obtain the exact values of these trials, so inclusion in the meta-analysis was not possible; however, these results suggest a possible positive effect of steroids on postoperative liver function, but yet without significant clinical impact.

There was a variety of different steroid schemes in the primary trials, of which the most common was a single dose of 500 mg methylprednisolone before surgery in four trials^27–29,47^. Two trials used hydrocortisone and continued the steroid administration until POD3 with stepwise dose reduction^30,51^. Of the remaining four trials, one applied a second intraoperative dose of 500 mg methylprednisolone^48^ and the others adapted the methylprednisolone group to the bodyweight of the patients, resulting in doses of more than 500 mg^31,49,50^. A direct comparison of different doses or preparations was not performed in any of the trials, thus the optimal intervention scheme cannot be finally clarified; however, as the subgroup of trials using methylprednisolone achieved statistical significance, whereas the subgroup of trials using hydrocortisone did not, methylprednisolone might be the preferred agent based on the current results. In the individual trials, the best effect was achieved in the trials with a single preoperative dose of 500 mg methylprednisolone. Thus, it seems unnecessary to continue the steroid administration after surgery. Until the optimal corticosteroid scheme is clarified in future studies, a single preoperative dose of 500 mg methylprednisolone may be recommended based on the present results.

A limitation of the present meta-analysis is that it was not possible to analyse the effect of perioperative steroid administration in specific subgroups, such as major *versus* minor liver resections or hepatic pedicle clamping *versus* no hepatic pedicle clamping; however, the primary trials did not report the results granularly enough to perform these analyses and individual patient data were not available. Looking at individual trials, two reported only major hepatectomies, of which one demonstrated a significant reduction of complications by steroid therapy^27^ and the effect in the second trial pointed into the same direction but without statistical significance^30^. On the other hand, in three trials more than 75 per cent of patients underwent minor liver resections and in each trial the rate of postoperative complications was similar between both groups without statistically significant differences^29,31,51^. This might suggest that the effect of perioperative steroid administration is more pronounced in patients undergoing major hepatectomies, but this issue needs further exploration. Regarding hepatic pedicle clamping, the Pringle manoeuvre was performed in the majority of cases in most trials included in the current meta-analysis. The trial by Schmidt *et al*.^49^ was the only one that did not apply the Pringle manoeuvre at all and in the trial by Bressan *et al*.^27^ it was performed in only 17.9 per cent of cases. While Bressan *et al*.^27^ demonstrated a significant reduction of postoperative complications in line with the results of the meta-analysis, Schmidt *et al*.^49^ did not find significant differences mainly owing to the small sample size. This prompts the assumption that the effect of perioperative steroid administration is similarly present in patients with and without hepatic pedicle clamping.

In addition to the above-mentioned benefits, it should be considered that steroids may be associated with numerous side effects. Among others, impaired wound healing, and the increased risk of infections due to suppression of the immune system, are discussed in the literature^35,36^. In addition, there are possible bleeding complications, for example due to gastrointestinal ulcers^37^ and a potential risk of thromboembolic events^55^. Thus, it seems necessary to investigate the influence of steroid administration on these postoperative complications to guarantee the safety of their administration. Unfortunately, only a limited number of primary trials reported these potential adverse effects and, for example, Bressan *et al*.^27^ reported a higher, albeit not statistically significant, rate of thromboembolic events in the steroid group. Thus, although no increased risk was detected in the present analysis, these results have to be interpreted in the light of the smaller sample size with limited statistical power; however, the existing literature demonstrating no increased risk for the occurrence of adverse effects with perioperative steroid administration corroborates the findings of our meta-analysis^35,56–58^.

In comparison with the previous systematic reviews on this topic by Hai *et al*.^52^ and Yang *et al*.^53^, the present meta-analysis included a substantially larger number of trials and patients, resulting in an increased power of the results. Hai *et al*.^52^ also found a reduction of overall complications, serum bilirubin, IL-6, and CRP in accordance to our results; however, despite the lower number of trials and patients in their analysis, they also included one non-randomized study^59^, which introduces potential bias and the critical appraisal was rather superficial. Yang *et al.*^53^ did not find a difference in postoperative complications, which is mainly attributable to the low power of the data due to the small study population. With regard to the laboratory parameters for inflammation and liver function, they found similar results, but some data were estimated from the graphs of the primary publications, causing a potential bias. Furthermore, the values of POD1 were used in all cases, which may be clinically less relevant than the postoperative peak values.

In conclusion, the current meta-analysis provides solid evidence that perioperative steroid administration reduces overall complications after elective liver resections. As there were no significant negative side effects and the number needed to treat was 10, it can be recommended routinely for clinical practice based on moderate certainty evidence assessed by the GRADE approach. Nonetheless, a multicentre confirmatory trial is warranted to corroborate the results of the present analysis and to finally ascertain the value of perioperative steroid administration in elective liver surgery.

## Supplementary Material

zrac139_Supplementary_Data

## Data Availability

As the present article is a systematic review and meta-analysis, all data analysed in this study are available in the published manuscripts of the included trials. Additional data and those computed by the analysis are available in the article and *Supplementary material*. If further information or data are requested, these can be shared upon reasonable request to the corresponding author.
