# Supplementary material for: Impact of perioperative steroid administration in patients undergoing elective liver resection: meta-analysis
Source: BJS Open. 2022 Dec 20;6(6):zrac139. doi: 10.1093/bjsopen/zrac139 (PMC9764439; doi:10.1093/bjsopen/zrac139)
Supplement: zrac139_Supplementary_Data [file zrac139_supplementary_data.docx]

**Impact of perioperative steroid administration in patients undergoing elective liver resection: meta-analysis**

Authors:

Laila Jötten^1^, Kira C. Steinkraus^1^, Benno Traub^1^, Sandra Graf^1^, André L. Mihaljevic^1^, Marko Kornmann^1^, Christoph W. Michalski^1^ and Felix J. Hüttner^1^

Department and Institution:

^1^Department of General and Visceral Surgery, Ulm University Hospital, Albert-Einstein-Allee 23, 89081 Ulm, Germany

Corresponding author:

Felix J. Hüttner

Department of General and Visceral Surgery

Ulm University Hospital

Albert-Einstein-Allee 23

89081 Ulm

Germany

Email: felix.huettner@uniklinik-ulm.de

**Supplementary Materials - Index**

| **Supplementary Appendixes** | |  |
| --- | --- | --- |
| Appendix S1: PubMed search strategy | | *pag. 3* |
| Appendix S2: Web of Science search strategy | | *pag. 3* |
| Appendix S3: Central search strategy | | *pag. 3* |
| **Supplementary Figures and Tables** | |  |
| Figure S1: Funnel plot for ‘overall complications’ | *pag. 4* | |
| Figure S2: Forest plot: SSI (organ/space) | *pag. 4* | |
| Figure S3: Forest plot: Mortality | *pag. 5* | |
| Figure S4: Forest plot: Pleural effusion | *pag. 5* | |
| Figure S5: Forest plot: Bleeding complications | *pag. 5* | |
| Figure S6: Forest plot: Reoperations | *pag. 6* | |
| Figure S7: Forest plot: Length of hospital stay (days) | *pag. 6* | |
| Figure S8: Forest plot: Duration of surgery (min) | *pag. 6* | |
| Figure S9: Forest plot: Intraoperative blood loss (ml) | *pag. 7* | |
| Figure S10: Forest plot: Postoperative peak AST (U/l) | *pag. 7* | |
| Figure S11: Forest plot: Postoperative peak ALT (U/l) | *pag. 7* | |

**Supplementary Appendixes**

**Appendix S1: PubMed search strategy**

"Hepatectomy"[Mesh] OR hemihepatectom*[tiab] OR hepatectom*[tiab] OR ((surgery[tiab] OR surgeries[tiab] OR resection*[tiab]) AND (liver[tiab] OR hepatic[tiab]))

AND

(cortisol*[tiab] OR hydrocortison*[tiab] OR "hydrocortisone acetate" [Supplementary Concept] OR "adrenal cortex hormones"[Mesh] OR (adrenal[tiab] AND cortex[tiab] AND hormone*[tiab]) OR corticoid*[tiab] OR glucocorticoid*[tiab] OR "Glucocorticoids" [Pharmacological Action] OR "steroids"[Mesh] OR steroid* OR methylprednisolon*[tiab] OR prednisolon*[tiab] OR prednison*[tiab])

AND

randomized controlled trial [pt] OR "controlled clinical trial"[Publication Type] OR randomized[tiab] OR placebo[tiab] OR "clinical trials as topic" [mesh: noexp] OR randomly [tiab] OR trial [ti]

NOT (animals [mh] NOT humans [mh])

**Appendix S2: Web of Science search strategy**

TS = (hemihepatectom* OR hepatectom*) OR TS= ((surgery OR surgeries OR resection*) NEAR (liver OR hepatic))

AND

TS= (cortisol* OR hydrocortison* OR corticoid* OR glucocorticoid* OR steroid* OR methylprednisolon* OR prednisolon* OR prednison*) OR TS= (adrenal NEAR cortex NEAR hormone*)

AND

TS = (random* OR “controlled clinical trial” OR placebo)

NOT (TI = rats OR mice OR mouse OR dogs OR cats)

**Appendix S3: Central search strategy**

MeSH descriptor: [Hepatectomy] explode all trees

OR (hemihepatectom* OR hepatectom*):ti,ab,kw OR ((surgery OR surgeries OR resection*) AND (liver OR hepatic)):ti,ab,kw

AND

(cortisol* OR hydrocortison* OR corticoid* OR glucocorticoid* OR steroid* OR methylprednisolon* OR prednisolon* OR prednison*):ti,ab,kw OR (adrenal AND cortex AND hormone*):ti,ab,kw

OR "steroids"[Mesh] OR "adrenal cortex hormones"[Mesh]

**Supplementary Figures and Tables**

**Figure S1: Funnel plot for ‘overall complications’**

**Figure S2: Forest plot: SSI (organ/space)**

**Figure S3: Forest plot: Pleural effusion**

**Figure S4: Forest plot: Reoperations**

**Figure S5: Forest plot: Mortality**

**Figure S6: Forest plot: Bleeding complications**

**Figure S7: Forest plot: Length of hospital stay (days)**

**Figure S8: Forest plot: Duration of surgery (min)**

**Figure S9: plot: Intraoperative blood loss (ml)**

**Figure S10: Forest plot: Postoperative peak AST (U/l)**

**Figure S11: Forest plot: Postoperative peak ALT (U/l)**
